# Diagnostic Value of Transthoracic Echocardiography in Patients with Coarctation of Aorta: The Chinese Experience in 53 Patients Studied between 2008 and 2012 in One Major Medical Center

**DOI:** 10.1371/journal.pone.0127399

**Published:** 2015-06-01

**Authors:** Zhenxing Sun, Tsung O. Cheng, Ling Li, Li Zhang, Xinfang Wang, Nianguo Dong, Qing Lv, Ke Li, Li Yuan, Jing Wang, Mingxing Xie

**Affiliations:** 1 Department of Ultrasound, Union Hospital, Tongji Medical College, Huazhong University of Science and Technology, Hubei Province Key Laboratory of Molecular Imaging, Wuhan, People’s Republic of China; 2 Department of Medicine, George Washington University Medical Center, 2150 Pennsylvania Avenue N. W., Washington, D. C., United States of America; 3 Department of Cardiovascular Surgery, Union Hospital, Tongji Medical College, Huazhong University of Science and Technology, Wuhan, People’s Republic of China; University of Cincinnati, College of Medicine, UNITED STATES

## Abstract

Although aortography is well known as the “gold standard” for the diagnosis of coarctation of aorta (CoA), the method is invasive, expensive and not readily accepted by some patients. Ultrasound diagnosis for CoA is non-invasive, inexpensive, readily accepted by every patient, and can be repeated as frequently as necessary. The purpose of this presentation is to evaluate the applicability of transthoracic echocardiography for the diagnosis of CoA. The echocardiographic appearances of 53 patients with CoA who had undergone surgery during a 5-year period from January 2008 to October 2012 were analyzed retrospectively, and the results were compared with findings at surgery. Fifty-three patients with CoA include six with isolated CoA and 47 of CoA associated with other cardiac anomalies. Of the 53 operated patients, 48 were correctly diagnosed preoperatively by echocardiography, while two were misdiagnosed as interrupted aortic arch and the diagnosis were missed in three other patients. Thus the diagnostic accuracy rate was 90.6%, and the misdiagnosis rate was 9.4%. Preoperative echocardiographic evaluation offers very satisfactory anatomic assessment in most patients with CoA. It makes preoperative angiography unnecessary. Thus transthoracic echocardiography should be the first-line method for the diagnosis of coarctation of the aorta.

## Introduction

Coarctation of the aorta (CoA) is a common congenital malformation. It may exist in isolation or with other congenital cardiovascular malformations. The clinical presentation depends on the location, the severity, and whether or not there are other associated cardiovascular malformations. Severe CoA usually causes heart failure. However, the prognosis is excellent if surgery is performed in time, especially in severe simple CoA [1]. Therefore, in order to increase survival rate and improve the patient’s quality of life, early diagnosis and prompt surgery are important.

The conventional method of diagnosis of CoA has been aortography. However, it is an invasive and expensive procedure with complications and radiation exposure. Echocardiography is a noninvasive method of examination, which can provide important information for the anatomic evaluation of the heart and major blood vessels as well as hemodynamics.

In this study, the echocardiographic appearances of 53 patients with CoA who had undergone surgery during the five-year period from January 2008 to October 2012 at Union Hospital were analyzed retrospectively to assess the value of echocardiography for the diagnosis of CoA.

## Methods

### Clinical Data (Study Population)

The study was approved by the local research ethics committee at Union hospital, Tongji medical college, Huazhong University of Science and Technology, China. All procedures were performed as part of routine care and testing, and not specifically for the purpose of this study. All data used were anonymized as all patients enrolled were identified by a progressive number. The individual in this manuscript has given written informed consent to publish these case details. All procedures and data analysis were performed by the authors; specific contributions of all enlisted authors are provided below.

**Fig 1:**
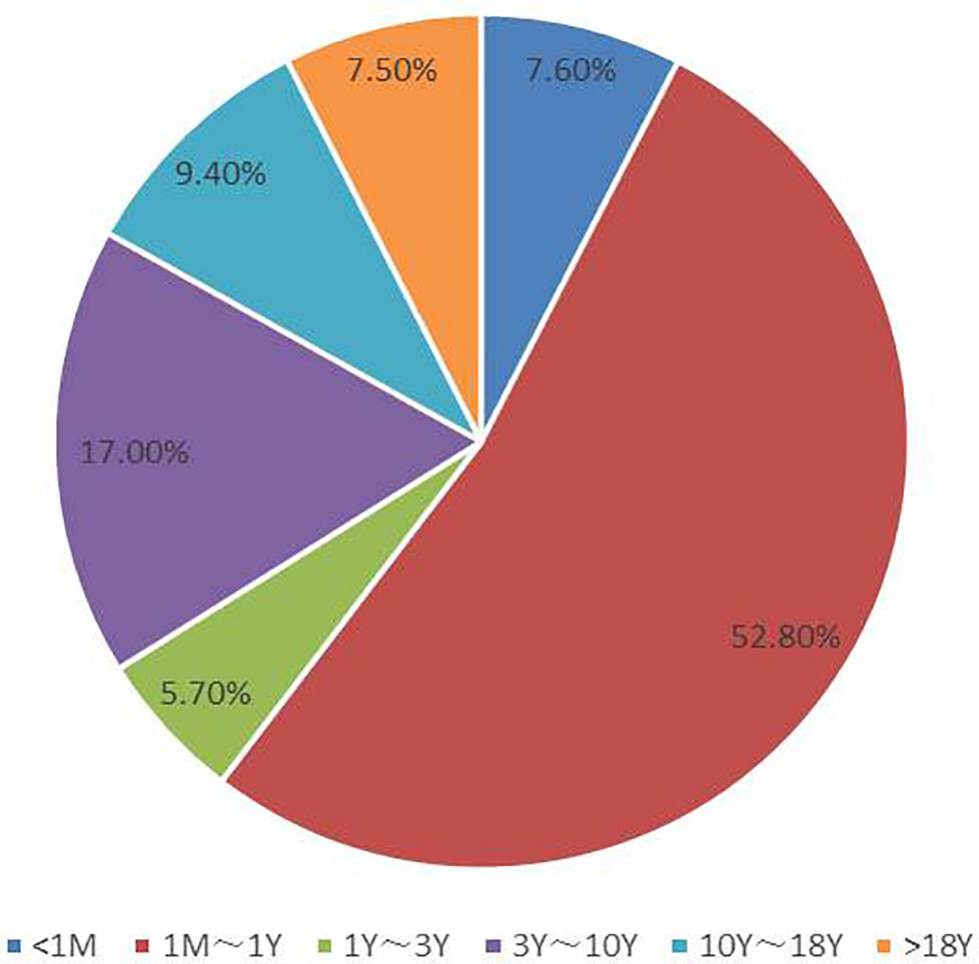
The age distribution of 53 patients with CoA.

The echocardiographic appearances of 53 patients with CoA who had undergone surgery from January 2008 to October 2012, were analyzed retrospectively. There were 38 males and 15 females; the median age was 0.63 years and the age distribution of 53 patients was presented in Fig 1. The clinical features of these patients were shown in Table 1. Twenty one patients had undergone other examinations such as aortography and/or computed tomographic angiography (CTA).

**Table 1:** Clinical features of 53 patients.

Clinical features	N(%)
Male/Female	38/15
Age range	4 days-52 years
Median age	7.5 months
Shortness of breath	53(100%)
History of heart failure and respiratory infection	18(33.9%)
Feeding difficulties	17(32.1%)
Hypertension	6(11.3%)
Cyanosis	4(7.5%)
Systolic murmur	41(77.4%)
Continuous murmur	12(22.6%)
Accentuation of second heart sound	29(54.7%)
Normal second heart sound	24(45.3%)
BP of upper limb higher than or equal to BP of lower limb	31(58.5%)

### Methodology

The Doppler ultrasonic diagnostic apparatus used was GE Vivid7 and Philips IE33 with 2.0–5.0MHz transducer. Patients were examined in left lateral position. The left ventricular long axis view, apical four-chamber view, large artery short axis view and suprasternal view were used in scanning with special attention to the structures of the atria, ventricles, aorta and their interconnections. The anatomical structure of the heart was probed carefully in left ventricular long axis view, large artery short axis view and apical four-chamber view so as to confirm whether or not there are other cardiovascular anomalies. The suprasternal views were used to observe the aortic arch, aortic arch branches and descending aorta in order to determine the location of the CoA and to measure the coarctation inner diameter and scope. Color Doppler was used to observe the blood flow and measure the maximum speed and differential pressure in the location of the CoA.

In this study, the accuracy rate of two-dimensional echocardiography, color Doppler and continuous wave Doppler for CoA diagnosis for 53 patients were intercompared. At the same time, the consistency comparison of the diagnosis of echocardiogram with CTA/Aortography and surgery were also analyzed.

### Statistical Analysis

Statistical analyses were performed using SPSS 19.0 software. Qualitative variables were expressed as percentage (%). Comparison was performed with the chi-square test or Fisher exact test for categorical data. For comparison of the accuracy rate of two-dimensional echocardiography, color Doppler and continuous wave Doppler, a p-value<0.0125 was considered statistically significant. For the consistency comparison of the diagnosis of echocardiogram with CTA/Aortography and surgery, a p-value<0.05 was considered statistically significant.

## Results

### Echocardiographic features of coarctation of aorta

**Fig 2:**
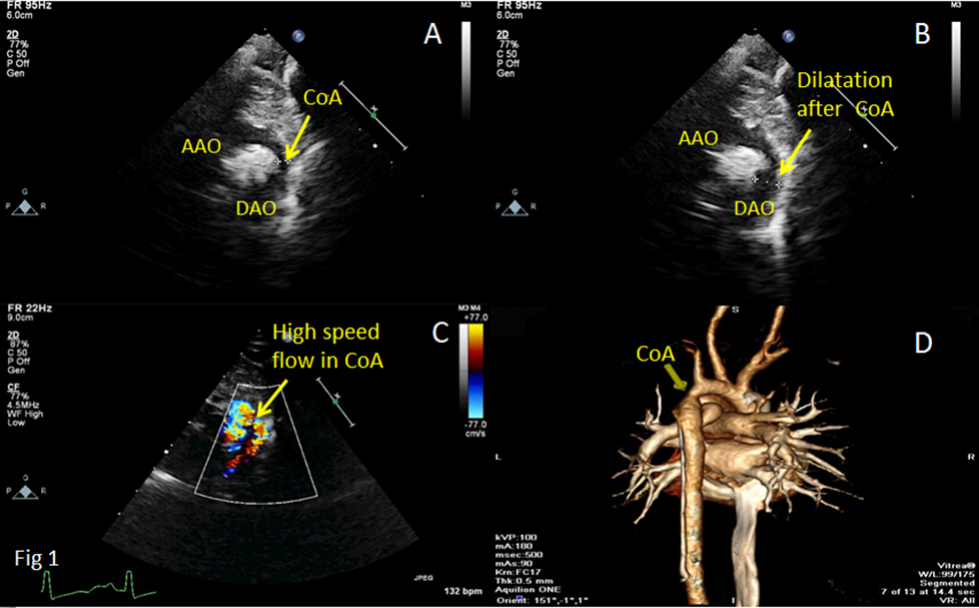
Transthoracic echocardiograms in suprasternal view of a 2-month-old boy showing[A] the location of the coarctation of aorta (CoA) between the ascending aorta (AAO) and descending aorta (DAO), [B] dilatation of the aorta after the CoA, and [C] high speed flow in the CoA. Computed tomographic angiography [D] obtained at 5 months of age confirmed the location of the CoA.

**Fig 3:**
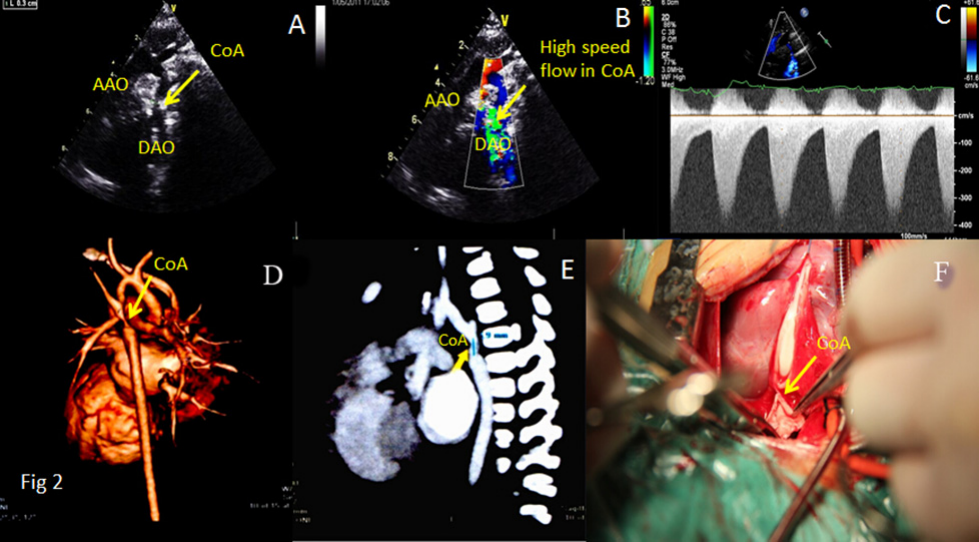
Transthoracic echocardiograms in suprasternal view of a 3-month-old girl showing [A] the location of the CoA, [B] the high flow signal in the CoA, and [C] characteristics of the CoA by continuous wave Doppler, including high speed jet spectrum during systole and extended speed reduction during diastole. Computed tomographic angiography [D,E] showed the location of the CoA. At surgery [F], the location of the CoA was confirmed. Abbreviations as in Fig 2.

**Fig 4:**
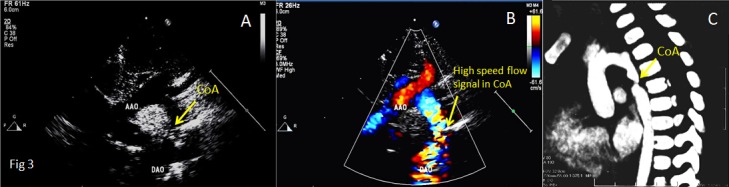
Transthoracic echocardiograms in suprasternal view of a 4-month-old boy showing [A] the location of the CoA between AAO and DAO and [B] the high speed flow signal in the CoA. Computed tomographic angiography [C] showed the location of the CoA. Abbreviations as in Fig 2.

**Fig 5:**
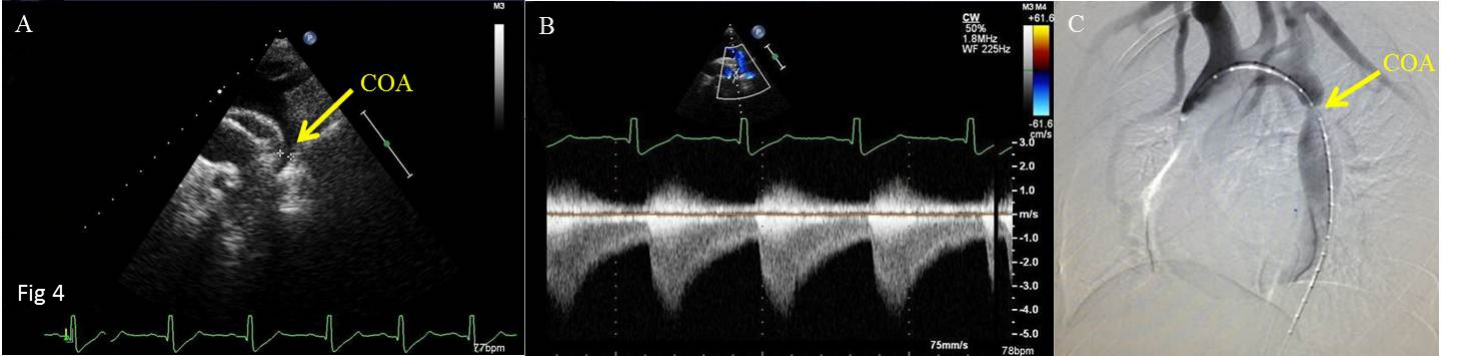
Transthoracic echocardiograms in suprasternal view of a19-year-old boy showing [A] the location of the CoA and [B]characteristics of the CoA by continuous wave Doppler, including high speed jet spectrum during systole and extended speed reduction during diastole. [C] Aortic angiography showed the location of the CoA. Abbreviations as in Fig 2.

Inaccordance with the relationship of the location of the coarctation with the ductus arteriosus, coarctation of the aorta is divided into coarctation of aorta-preductal type and coarctation of aorta-postductal type. Echocardiographic features of coarctation of aorta are as follows: (I) In coarctation of aorta-preductal type, the inner diameters of distal aortic arch and aortic isthmus narrow significantly, and the aortic arch and/or descending aorta reveal an irregular stricture. In coarctation of aorta-postductal type, the descending aortic constriction located distal to the left subclavian artery presents a gourd-shaped appearance (Figs 2A, 3A, 4A and 5A). Whether the coarctation of aorta is of the preductal or postductal type, the distal descending aorta reveals dilatation after the coarctation (S1 Fig). Of the 36 coarctation patients diagnosed by two-dimensional echocardiography, 4 are of preductal type (the narrowest diameters range from 0.2cm to 0.5cm) and 32are of postductal type(the narrowest diameters range from 0.3cm to 0.7cm). The accuracy rate by two-dimensional echocardiography was 67.9%. (II) Color Doppler echocardiograms in both the preductal and postductal types revealed dim blood flow signal before the coarctation of aortic arch and aortic isthmus and multi-colored high flow signal after the coarctation. The blood flow signal became thin at the coarctation and presents diffused images when it passed the coarctation (Figs 2C, 3B and 4B; S2, S3 and S4 Figs). Of the 53 patients, 47 had been diagnosed by color Doppler ultrasound, including 5 of coarctation of aorta-preductal type and 42 of coarctation of aorta-postductal type. The accuracy rate by color Doppler ultrasound was 88.7%. (III) Spectral Doppler: continuous wave Doppler characteristics of CoA are high speed jet spectrum during systole, retroposed peak spectral frequency and extended ejection time (Figs 3C and 5B). The high blood flow speed ranges were 2.4m/s to 4.9m/s and the maximum differential pressures were 25mmHg to 98mmHg. Of the 53 patients, 48 had been diagnosed by color Doppler ultrasound, including 5 of CoA of preductal type and 43 of CoA of postductal type. The accuracy rate by continuous wave Doppler ultrasound was 90.6%. (IV) Other findings comprised left ventricular hypertrophy, dilated ascendingaorta, and intracardiac malformations including atrial septal defect (ASD), ventricular septal defect (VSD), patent ductus arteriosus (PDA) and anomalies of the aortic valve.

The diagnostic accuracy by two-dimensional echocardiography showed a significant difference from color Doppler echocardiography and spectral Doppler. However there was no significant difference between color Doppler echocardiography and spectral Doppler (Table 2).

**Table 2:** Comparison of correct diagnosis rate by two-dimensional ultrasound, color Doppler ultrasound and spectral Doppler.

Group	Correct Diagnosis	Misdiagnosis	Total	$\chi^{2}$	p*
Two dimensional ultrasound	36	17	53		
Color Doppler ultrasound	47	6	53	6.72	0.01
Total	83	23	106		
Two dimensional ultrasound	36	17	53		
Continuous wave Doppler	48	5	53	8.55	0.004
Total	84	22	106		
Color Doppler ultrasound	47	6	53		
continuous wave Doppler	48	5	53	0.10	0.75
Total	95	11	106		

* p-value<0.0125 was considered statistically significant

### Comparison of diagnoses with transthoracic echocardiography (TTE) and with computed tomographic angiography (CTA) and/or Aortography

Of the 21 among the 53 patients, including 2 of CoA-preductal type and 19 of CoA-postductal type, diagnosis with CTA and/or Aortography(Figs 2D, 3D, 3E,4C and 5C) were in line with findings at surgery (Fig 3F). 19 patients, including 2 of CoA-preductal type and 17 of CoA-postductal type were diagnosed by echocardiography and two were misdiagnosed. The diagnostic accuracyrate was 90.5%. There was no significant difference in diagnostic accuracy between TTE and CTA/Aortography(p = 0.456) (Table 3).

**Table 3:** Comparison of correct diagnosis rate between TTE and CTA and/ or Aortography.

Groups	Correct Diagnosis	Misdiagnosis	Total	Accuracy rate
TTE	19	2	21	90.5%
CTA and/or Aortography	21	0	21	100%
Total	40	2	42	95.2%

TTE: transthoracic echocardiography; CTA: computed tomographic angiography

### Findings at surgery

At surgery (Fig 3F), the CoA was classified into coarctation of solitary type and coarctation combined with other intracardiac malformations. Of the 53 patients with CoA, 6 were of the solitary type and 47 combined with other intracardiac malformations. CoA was preductal in 5 and postductal in 48 patients (Table 4). Fifty-three patients were examined by senior ultrasound doctors with transthoracic echocardiography before surgery, and in 48 (90.6%) the diagnosis was in line with surgical findings. There was no significant difference in diagnostic accuracy rate (p = 0.067) between findings with TTE and surgery (Table 5).

**Table 4:** Surgical findings in 53 patients.

Types	N (%)
Coarctation of solitary type	6(11.3%)
Coarctation with other intracardiac malformations	47(88.7%)
Coarctation of aorta-preductal type	5(9.4%)
Coarctation of aorta-postductal type	48(90.6%)
Associated findings	
LVH	18(34.0%)
DSA	26(49.1%)
DPA and PAH	31(58.5%)
Associated intracardiac malformations^#^	
ASD	19(35.8%)
VSD	23(43.4%)
PDA	29(54.7%)
CCHD	4(7.5%)
BAVD	8(15.1%)
UAVD	1(1.9%)
TAPVD	1(1.9%)
NVM	3(5.7%)
CORPA	2(3.8%)
LSVC	5(9.4%)
MVD	3(5.7%)

^#^some patients have several intracardiac malformations

LVH: left ventricular hypertrophy; DSA: dilated ascending aorta; DPA: Dilated pulmonary artery; PAH: pulmonary arterial hypertension; ASD: atrial septal defect; VSD: ventricular septal defect; PDA: patent ductus arteriosus; CCHD: combined congenital heart disease; BAVD: Bicuspid aortic valve disease; UAVD: unicuspid aortic valve disease; TAPVD: total anomalous pulmonary venous drainage; NVM: noncompaction of ventricular myocardium; CORPA: Coarctation of right pulmonary artery; LSVC: left superior vena cava; MVD: Mitral valve deformity.

**Table 5:** Comparison of findings at TTE and surgery.

Groups	Correct Diagnosis	Misdiagnosis	Total	Accuracy rate
TTE	48	5	53	90.6%
Surgery	53	0	53	100%
Total	101	5	106	95.3%

TTE: transthoracic echocardiography

## Discussion

In this study, we introduced Echocardiographic features of coarctation of aorta and compared the accuracy rate of two-dimensional echocardiography, color Doppler and continuous wave Doppler. Then the consistency comparison of the diagnosis of echocardiogram with CTA/Aortography was analyzed. The operation findings were listed and compared with echocardiographic results.

### Incidence of CoA

Early detection and treatment of coarctation of aorta (CoA) is extremely important [1]. CoA accounts for 6–8% of live births with congenital heart disease[2] and has an estimated incidence of 1 in 2,500 births [3, 4].It is likely that the incidence is higher in stillborn babies [5]. There are more male patients than female patients with CoA, with a reported ratio of male-to-female between 1.27:1 and1.74:1 [6].Our series of 53 patients included 38 males and 15 females, with a sex ratio of male to female equal to 2.53:1, which was roughly identical to those reported in the literature [6].

### Anatomical features of CoA

CoA is a localized constriction of the aorta, most frequently located near the insertion of ductus arteriosus or ligamentum arteriosum. According to the location of CoA, CoA is divided into the pre-ductal type and post-ductal type, with the post-ductal type being more common. In our study, 90.6% was of the post-ductal type and 9.4% of the pre-ductal type. CoA can exist either in isolation or more commonly in association with other congenital heart defects, such as patent ductus arteriosus (PDA), atrial septal defect (ASD), ventricular septal defect (VSD), total anomalous pulmonary venous drainage (TAPVD), bicuspid aortic valve and mitral valve diseases. The reported incidence of associated cardiac abnormalities was 52.3%-74.1% [7,8]. In our series, there were 6 patients of CoA of solitary type and 47 patients with other intracardiac malformations, including, in descending order of frequency, PDA (54.7%), VSD (43.4%), ASD (35.8%) and TAPVD (1.9%).The incidence rate of associated cardiac abnormalities is in line with those reported in the literature [7,8].

### Imaging diagnosis of CoA

In the past the diagnosis of CoA relied on clinical symptoms and physical examination. The typical clinical features of CoA include higher blood pressure in upper limb than that in the lower limb, enhanced radial artery pulsation and weakened femoral artery pulsation. However, not all CoA patients present the above-mentioned clinical features [9,10]. Resistant hypertension was the only symptom in some patients whose blood pressure of upper limb was not higher than blood pressure of lower limb and the diagnosis could be easily missed if one relied on the difference of blood pressure between the upper and lower limbs [11].

Angiography has been the recognized “gold standard” of diagnosing CoA [12]. It can show the location, range and degree of CoA as well as the anatomical features of collateral circulation. The development of ascending aorta and aortic arch, its branches and other cardiac defects can be clearly seen on aortography [13]. However, the method is invasive, expensive and not readily accepted by some patients. Furthermore, it exposes both the patient and the operator to irradiation. In addition, if the operator employs the retrograde approach, the catheter may not traverse the narrow segment of the aorta easily, and the vascular complication rate is quite high when the inner diameter of the coarctated segment is very narrow. Electron beam CT and magnetic resonance imaging have also been employed in recent years with very good diagnostic accuracy, but the post-processing of images is quite complex, rather time-consuming and very expensive.

Transthoracic echocardiography is non-invasive, inexpensive, and easily repeatable. Besides, it not only reveals the precise anatomy of CoA, but also provides much information concerning other cardiovascular structures, cardiac function and hemodynamics. Hence, color echocardiography should be a primary method for diagnosis of CoA [14,15], especially for pregnant women to avoid irradiation [16]. The echocardiographic diagnosis of 48 of our 53 patients was in line with findings at surgery; the accuracy rate was 90.6%, which is consistent with those reported in the literature [17,18].

Echocardiography is also very useful in the postoperative evaluation of patients with CoA. The life expectancy of patients with CoA has been shown to decrease even though the operation has been successful [19,20]. The postoperative patients of CoA are still at risk for such complications as persistent hypertension and complications of chronic hypertension including coronary heart disease, myocardial infarction, stroke, dissecting aneurysm and early deaths [19]. Furthermore, persistent hypertension results in left ventricular hypertrophy and reduced left ventricular diastolic function [19,20]. Resting hypertension persists in 10–30% of patients following surgical repair, and exercise may induce hypertension in 30–65% of such patients [20]. However, exercise test may not be suitable for young children; pharmacological stress test must be employed for this reason. Banaszaketal [21] suggested dobutamine stress echocardiography for evaluation of the effects of surgical repair of CoA in children. Echo- cardiography is also very valuable in detecting postoperative re-coarctation [22–24], aortic aneurysm formation at the site of repair [25], and pulmonary hypertension [26].

Transthoracic echocardiography also has diagnostic value on other congenital heart diseases, including sinus of valsalva aneurysm [27], coronary sinus septal defect [28], coronary artery fistula [29], left coronary artery arising from the right pulmonary artery [30] and so on excerpt aortic coarctation.

### Echocardiographic misdiagnosis and missed diagnosis

Echocardiography has a few limitations in measuring the length of the CoA and visualizing collateral circulation [18].Two patients in our series were misdiagnosed as interruption of aortic arch because their sections of CoA were very long. So these patients should have a CT scan if necessary.

Compared with the CoA of preductal type, CoA of postductal type was more difficult to diagnose because of the long coarctation sections, blocked air and poor acoustic window. So the CoA of the postductal type was easily missed in diagnosis. Compared with color Doppler and continuous wave Doppler, 2D echocardiography alone not always sufficient to diagnose CoA readily and accurately. Only by incorporating all three modalities, the accuracy rate would be greatly enhanced as shown in Table 2. Finally, the diagnostic accuracy by TTE in our series of patients with CoA was 90.5% as compared with that by CTA, which was 100% (Table 3).

## Conclusions

Transthoracic echocardiography with color Doppler is the contemporary cornerstone for diagnostic assessment of patients with coarctation of aorta [31]. It is non-invasive, direct, safe, fast, inexpensive and readily repeatable. It is also useful for quantification of left ventricular mass [26,32] and exclusion of other intracardiac abnormalitie [32]. It can provide much more information of cardiovascular structures, cardiac function and hemodynamics compared with angiocardiography. Finally, CoA being one of the most costly conditions for pediatric hospitalizations [33], echocardiography certainly should be the first-line method for the diagnosis of CoA, both preoperatively and postoperatively, from the economical point of view in the current era of escalating health care cost.

## Supporting Information

S1 Fig Transthoracic echocardiograms in suprasternal view of a 2-month-old boy showing. The location of the coarctation of aorta (CoA) between the ascending aorta (AAO) and descending aorta (DAO).(AVI).
(AVI)

S2 Fig Transthoracic echocardiograms in suprasternal view of a 2-month-old boy showing. High speed flow in the CoA.(AVI).
(AVI)

S3 Fig Transthoracic echocardiograms in suprasternal view of a 3-month-old girl showing. The high flow signal in the CoA.(AVI).
(AVI)

S4 Fig Transthoracic echocardiograms in suprasternal view of a 4-month-old boy showing. The high speed flow signal in the CoA.(AVI).
(AVI)
